# Integrated Microbiome–Metabolome Analysis Reveals Stage-Dependent Alterations in Bacterial Degradation of Aromatics in *Leptinotarsa decemlineata*

**DOI:** 10.3389/fphys.2021.739800

**Published:** 2021-09-30

**Authors:** Wei-Nan Kang, Lin Jin, Hong-Yu Ma, Guo-Qing Li

**Affiliations:** ^1^Education Ministry Key Laboratory of Integrated Management of Crop Diseases and Pests, College of Plant Protection, Nanjing Agricultural University, Nanjing, China; ^2^Public Laboratory Platform, College of Plant Protection, Nanjing Agricultural University, Nanjing, China

**Keywords:** *Leptinotarsa decemlineata*, stage-specific bacteria, habitat, aromatics, biodegradation

## Abstract

To avoid potential harm during pupation, the Colorado potato beetle *Leptinotarsa decemlineata* lives in two different habitats throughout its developmental excursion, with the larva and adult settling on potato plants and the pupa in soil. Potato plants and agricultural soil contain a specific subset of aromatics. In the present study, we intended to determine whether the stage-specific bacterial flora plays a role in the catabolism of aromatics in *L. decemlineata*. Kyoto Encyclopedia of Genes and Genomes (KEGG) pathway analysis of the operational taxonomic units (OTUs) obtained by sequencing of culture-independent 16S rRNA region enriched a group of bacterial genes involved in the elimination of mono- and polycyclic aromatics at the pupal stage compared with those at the larval and adult periods. Consistently, metabolome analysis revealed that dozens of monoaromatics such as styrene, benzoates, and phenols, polycyclic aromatics, for instance, naphthalene and steroids, were more abundant in the pupal sample. Moreover, a total of seven active pathways were uncovered in the pupal specimen. These ways were associated with the biodegradation of benzoate, 4-methoxybenzoate, fluorobenzoates, styrene, vanillin, benzamide, and naphthalene. In addition, the metabolomic profiles and the catabolism abilities were significantly different in the pupae where their bacteria were removed by a mixture of three antibiotics. Therefore, our data suggested the stage-dependent alterations in bacterial breakdown of aromatics in *L. decemlineata*.

## Introduction

For holometabolous insect species, sessile pupae are defenseless against potentially harmful factors such as pathogen infection, parasitism, predation, and desiccation. Consequently, a lot of Holometabolans leave their host plants and pupate in soil, an adaptation termed as ontogenetic niche shift (ONS). For example, the final instar larval period of the Colorado potato beetle *Leptinotarsa decemlineata* is divided into two subphases, the feeding and wandering stages. Whereas, a feeding beetle continuously gnaws potato foliage, a wandering larva typically undergoes an ONS to pupate in the soil (Meng et al., 2019). Obviously, a *L. decemlineata* beetle lives in two different habitats throughout its developmental excursion, with the larva and adult settling on potato plants and the pupa in soil.

In the present paper, we focused on a serious challenge for *L. decemlineata*: degrading excessive aromatics derived from potato plants and agricultural soil. In potato plants, the metabolism of three aromatic amino acids (i.e., tyrosine, phenylalanine, and tryptophan) can produce large number of monoaromatics. These monoaromatics can be used to biosynthesize structurally complex substances. For instance, catecholamines, derived from tyrosine and phenylalanine, can be used to produce betalains, alkaloids, melanins, and hydroxycinnamic acid amides (Gandia-Herrero and Garcia-Carmona, 2013; Kostyn et al., 2020). To improve soil quality and increase crop yield, the plant residues, such as straws and stubbles after harvest, organic fertilizers, for example, manure and human excreta, and other organic landfills, are often applied to agricultural fields. These additives generate a variety of organic compounds incorporated into agricultural soil (Kumar and Goh, 2000; Hanselman et al., 2003; Kjaer et al., 2007). Among these organic pollutants are monoaromatics, for instance, benzene, toluene, ethylbenzene, xylene (collectively known as BTEX), styrene, and phenol and polycyclic aromatics such as naphthalene, dioxin, and steroids (Chen et al., 2017; Steinmetz et al., 2019).

Some aromatics are toxic, antinutritive, or/and repellent at high concentrations (Ceja-Navarro et al., 2015; Hammer and Bowers, 2015; Vilanova et al., 2016; Berasategui et al., 2017). Specifically, catecholamines are toxic and can pose a threat to cellular components (Kostyn et al., 2020). Moreover, quinones can lead to the formation of quinoprotein by a reaction between dopaquinone and the sulfhydryl groups of proteins and cause negative effects such as enzyme deactivation, mitochondrial dysfunction, DNA fragmentation, and apoptosis (Mushtaq et al., 2013; Kostyn et al., 2020). Furthermore, dopa may be incorporated into proteins *via* mimicking tyrosine or phenylalanine in the respective tRNA synthesis (Rodgers and Shiozawa, 2008), or serves as a deterrent to herbivores (Fürstenberg-Hägg et al., 2013; Kostyn et al., 2020). Therefore, these aromatics may adversely affect *L. decemlineata* when accumulated to high concentrations in the body.

It is well-known that insects have evolved different mechanisms to circumvent deleterious effects from the environment (Berasategui et al., 2017). One of the strategies is enlisting the cooperation of bacteria (Lesperance and Broderick, 2020). These bacteria dramatically change during development (Chen et al., 2016; Kang et al., 2021). For instance, a shift of microbial flora coupled with ONS has been documented in *L. decemlineata*, where a total of 18 bacteria genera are specifically distributed in pupae. In contrast, a subset of bacteria genera has larger populations in larvae and adults than those in pupae (Kang et al., 2021). Accordingly, we hypothesized that the stage-specific bacteria flora forms different symbiotic interplays with *L. decemlineata* to biodegrade stage-dependent aromatics from potato plant and agricultural soil, respectively.

The objective of our study was to test the hypothesis. First, we found that a group of bacterial genes involved in the catabolism of monoaromatics and polycyclic aromatics were abundantly expressed at the pupal stages. Second, we evaluated the metabolome profiles in the fourth-instar larvae, pupae, and adults by ultra-performance liquid chromatography–quadrupole–time of flight mass spectrometry (UPLC-Q-TOF MS). We identified stage-specific aromatic biomarkers and active pathways. Finally, we removed bacteria in the pupae by a mixture of three antibiotics, and compared the metabolome profiles between control and treated pupae. Our results revealed stage-dependent alterations in the bacterial breakdown of aromatics in *L. decemlineata*. We argue that the larvae and adults rely on two routes to deal with excessive aromatics, namely, bacterial biodegradation and intestinal excretion. In contrast, the pupae mainly depend on bacteria to catabolize aromatics since the alimentary canal is not well-developed.

## Methods and Materials

### Insect Rearing and Sampling

The *L. decemlineata* beetles were routinely reared using a previously described method (Meng et al., 2019). Briefly, the beetles were maintained in an insectary at 28°C, under a 16:8 h (light/dark) photoperiod, and 50–60% relative humidity using potato foliage at the vegetative growth or young tuber stages to assure sufficient nutrition. At this feeding protocol, the larvae progressed the first-, second-, penultimate-, and final-instar stages with approximate periods of 2, 2, 2, and 4 days, respectively. On reaching full size, the final larval instars stopped feeding, dropped to the ground, burrowed to the soil, and entered the prepupal stage. The prepupae spent ~3 days to pupate. The pupae lasted about 5 days and the adults emerged.

All solvents used for UPLC-Q-TOF-MS analysis were of analytical or HPLC grade and purchased from Sigma-Aldrich Co., Ltd. (Shanghai, China).

The detailed procedure of sample preparation for UPLC-Q-TOF-MS was as follows: ten (5 males and 5 females) 2-day-old fourth-instar larvae, ten 4-day-old pupae, and ten 5-day-old adults were collected as a replicate. The collection continued for three consecutive generations to generate three biologically independent replicates. The specimens were individually weighed and then crushed in a pre-chilled mortar using a pellet pestle. Each sample was lyophilized, added with 1 ml of methanol/water (7:3, v/v), vortexed, and sonicated twice on ice for 30 min. Then the metabolite was incubated at −20°C for 1 h and centrifuged at 13,000 rpm at 4°C for 15 min. The supernatant was lyophilized and stored at −80°C.

### Analysis for Operational Taxonomic Units Data From High-Throughput Sequencing of 16S rRNA Genes

Functional prediction was carried out based on the operational taxonomic units (OTU) data downloaded from the NCBI Sequence Read Archive (SRA) database (Accession Number: PRJNA613266) (Kang et al., 2021). The OTU data were obtained by high-throughput sequencing of 16S rRNA genes from three biologically independent collections from three consecutive generations. Each collection included ten 2-day-old fourth-instar larvae, ten 4-day-old pupae, and ten 5-day-old adults with the sex ratio of 1:1 (5 males and 5 females). The PICRUSt algorithm was used to infer the functions of the bacterial communities through the Kyoto Encyclopedia of Genes and Genomes (KEGG) database (Kanehisa et al., 2012).

### Analysis Conditions of UPLC/Q-TOF-MS

The supernatant was reconstituted with 500 μl of methanol, vortexed, and centrifuged at 20,000 rpm at 4°C for 20 min. Then the supernatant was filtered through a 0.22 μm (nylon) syringe filter and analyzed by UPLC-Q-TOF-MS system (Waters). Each sample was analyzed six times (i.e., 2 μl aliquot of each sample was injected six times).

Since the retention times or even elution order in analytical system may vary during the UPLC-Q-TOF-MS analysis, it is necessary to monitor the system consistency. In this study, a 60 μl mixture of the control (30 μl) and treatment samples (30 μl) was used as a quality control (QC) sample for method validation. A QC sample ran four times prior to beginning the whole sample list. Moreover, a QC sample was run every six samples during the analytical run.

The analysis was performed on an HSS T3 column (2.1 × 100 mm, 1.8 μm; Waters Acquity), using an ACQUITY UPLC I-Class PLUS System (Waters) coupled with a Xevo® G2-XS QT of High-Definition Mass Spectrometer (Waters). Mobile phase A was water and mobile phase B was 0.1% formic acid acetonitrile solution. The gradient elution procedure was set as follows: 0–1 min, 0–2% B; 1–2 min, 2–25% B; 2–4 min, 25–60% B; 4–7.5 min, 60–90% B; 7.5–9.5 min, 90–99% B; 9.5–12.5 min, 99% B; 12.5–13 min, 99–2% B; and 13–16 min, 2% B. The flow rate was 0.4 ml/min, and the column temperatures were held constant at 45°C.

Each sample was detected by positive and negative ion modes using an electrospray ionization mass spectrometer (ESI-MS). The product ion scan was acquired using the first- and second-level mass spectrometry data acquisition method based on the Photodiode Array (PDA) detector.

The ESI conditions were as follows: nitrogen was used as cone gas and desolvation gas at a flow rate of 50 and 800 l/h, respectively. The source temperature was 120°C and desolvation gas temperature was 450°C. Quality scanning range was set *m*/*z* 50–1,200. In positive ion mode, capillary, cone, and extraction cone voltages were 3.0 kV, 40 V, and 5.0 V, respectively. In negative ion mode, capillary, cone, and extraction cone voltages were 2.0 kV, 40 V, and 5.0 V, respectively. MS data were acquired in full-scan mode from 100 to 1,000 Da.

### Data Processing and Statistical Analysis of UPLC/Q-TOF-MS Data

The raw data detected by UPLC-Q-TOF-MS were loaded on the commercial metabolites database Progenesis QI (Waters Corporation, Milford, USA) for peak detection, alignment, and normalization, as well as the main information, such as the mass, retention time, and intensity of the peaks in each chromatogram. The metabolites were identified by comparing their retention times, *m*/*z* values, and MS fragmentation patterns with those of commercial standard compounds. Fragmentation patterns collected in online databases, such as MycompoundID (http://www.mycompoundid.org), MassBank (http://www.massbank.jp), ChemSpider database (www.chemspider.com), and METLIN (http://metlin.scripps.edu) were also considered, especially when no authentic standard compounds were available.

Before multidimensional statistical analysis, the data were processed: the missing values of the original data >50% were excluded. The processed data were then imported into SIMCA-P14.1 software (Umetrics, Umea, Sweden) for pattern recognition, and Pareto scaling was used to preprocess the data for principal component analysis (PCA) and orthogonal PLS-DA analysis (OPLS-DA). According to the Variable Importance for the Projection (VIP) obtained by the OPLS-DA model and Max Fold Change (MFC) from Progenesis QI, the influence intensity and explanatory ability of each metabolite on the classification and discrimination of each group of samples were evaluated, and the biologically significant differential metabolites were mined. The larger the VIP and MFC values, the greater the contribution of the metabolite in the differentiation of the sample, and the variable with VIP > 1 and MFC > 2 was generally considered to have a significant difference. In the experiment, based on the screening criteria of VIP > 1 and MFC > 2, the substances between the groups were initially screened. Next, the univariate statistical analysis was used to verify whether the selected metabolites had significant differences. Ions meeting VIP > 1, MFC > 2, and 0.05 < *p* < 0.1 were considered differential metabolites; VIP > 1, MFC > 2, and *p* < 0.05 were regarded as significantly different metabolites.

To highlight differential biomarker role, the resulting significant differential metabolites were analyzed in KEGG (http://www.kegg.jp) to resolve the topological trait of metabolic pathways.

### Antibiotics Exposure and Examination of Bacteria in Resultant Beetles

The same method was used to expose the larvae to an antibiotic mixture (Löfmark et al., 2010; Xia et al., 2020). Briefly, a mixture containing three antibiotics (1 mg/ml ciprofloxacin, 1 mg/ml levofloxacin, and 2 mg/ml metronidazole) in 1% Tween 20 aqueous solution was used to immerse potato foliage. Tween 20 in sterile water (1%) was set as the control group. Ten newly ecdysed fourth-instar larvae were confined in a Petri dish (9 cm diameter and 1.5 cm height) containing five treated leaves. The larvae were allowed to feed on the treated leaves until they reached the prepupal stage. The foliage was replaced with freshly treated ones each day.

The total microbial DNAs were individually extracted from 4-day-old pupae having fed on the antibiotic mixture or control solution as larvae, using E.Z.N.A.® Tissue DNA Kit (Omega). The removal of bacteria was examined using a pair of universal primers, the forward primer 5′-TCCTACGGGAGGCAGCAGT-3′ and reverse primer 5′-GGACTACCAGGGTATCTATCCTGTT-3′, of 16S rDNA from the Domain Bacteria (Nadkarni et al., 2002; Silkie and Nelson, 2009). Quantitative DNA measurements were performed by real-time quantitative reverse transcription PCR (qRT-PCR) in technical triplicate. Relative expression level of 16S rDNA was calculated by the 2^−Δ*ΔCT*^ method, using the geometric mean of two internal control genes (*LdRP4* and *LdARF1*) (Shi et al., 2013) for normalization.

To further examine the removal of bacteria, the dissected guts from newly emerged adults in control and treatment groups were homogenized. The supernatants separated by centrifugation at 500 g were spread on a plate culture medium (Luria–Bertani) after diluting for 10 times. The dishes were inoculated at 30°C for 17 h and then the bacterial spots were detected.

The metabolites in control and bacteria-removed pupae were tested using UPLC-Q-TOF-MS system and the data were analyzed using the method described above.

### Statistical Analysis

Using SPSS for Windows (Chicago, IL, USA), one-way analysis of variance (ANOVA) with a Tukey–Kramer test, or Student's *t*-test was performed to determine significant difference between average values (±SD). Results were considered statistically significant when *p* < 0.05.

## Results

### Functional Analysis of Bacteria

**Figure 1:**
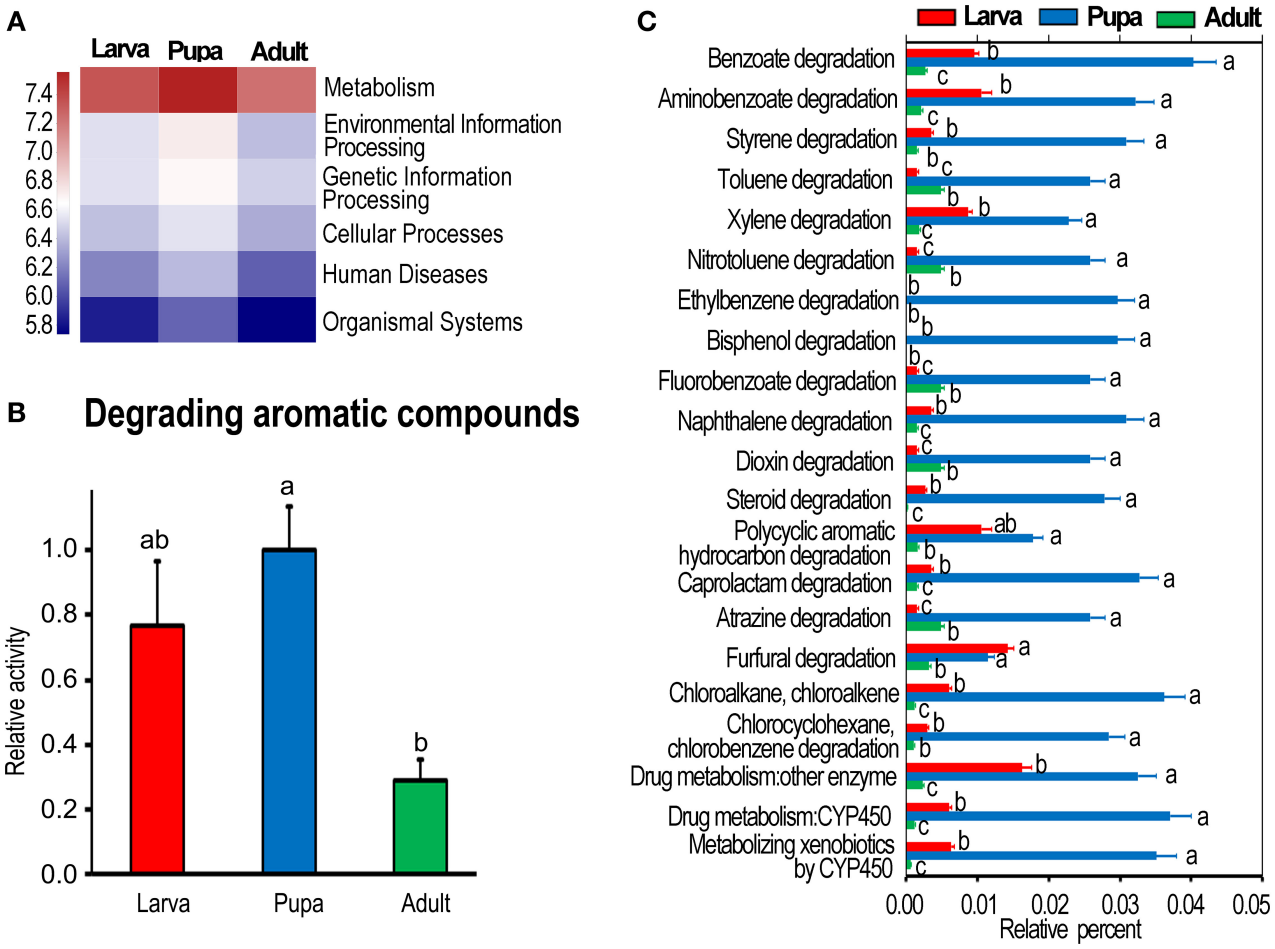
Kyoto Encyclopedia of Genes and Genomes (KEGG) analysis of bacterial flora in *Leptinotarsa decemlineata*. **(A)** Shows whole metabolism activities at KEGG level 1. The relative activities in **(B)** are the ratios of relative percentages in larvae and adults relative to that in the pupae, which is set as 1. **(C)** Displays the relative activity (percentage) of individual aromatics in the larvae, pupae, and adults at the third KEGG level. The columns represent averages with vertical lines indicating SE. Different letters indicate a significant difference at *p-*value < 0.05 using analysis of variance with the Tukey–Kramer test.

The OTUs of bacteria obtained by PCR amplification and sequencing of culture-independent 16S rRNA (Kang et al., 2021) were analyzed using KEGG enrichment analysis. At KEGG level 1, metabolism was the most active one, especially in the pupa group (Figure 1A). The highly expressed genes associated with the catabolism of aromatic compounds (at KEGG level 3) were observed in the pupa group, followed by those in the larvae, and the levels were lower in the adult collection. One-way ANOVA revealed that a statistical significant difference was present between pupa and adult groups (*p* < 0.05) (Figure 1B).

Correspondingly, the actively expressed genes (at KEGG level 3) involved in the degradation of benzoate, aminobenzoate, styrene, toluene, xylene, nitrotoluene, ethylbenzene, bisphenol, fluorobenzoate, naphthalene, dioxin, steroid, polycyclic aromatic hydrocarbon, caprolactam, atrazine, furfural, chloroalkane, chloroalkene, chlorocyclohexane, and chlorobenzene were significantly richer in the pupa group than those in the larva and adult collections (Figure 1C).

### Potential Biomarkers of Aromatic Compounds

**Figure 2:**
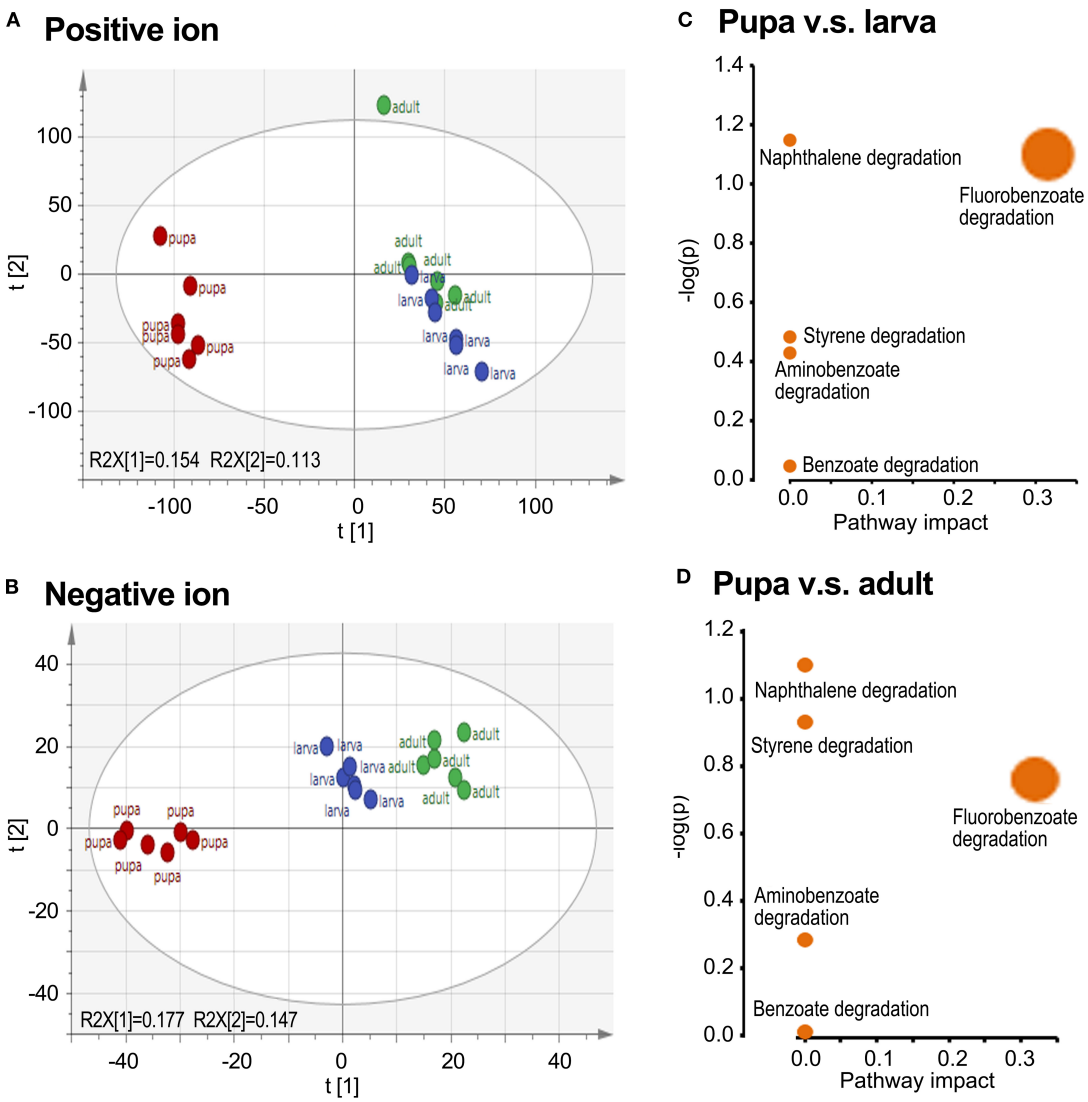
Grouping comparison of metabolomics in larvae, pupae, and adults in *L. decemlineata*. The ultra-performance liquid chromatography–quadrupole–time of flight mass spectrometry (UPLC-Q-TOF-MS) data were analyzed by principal component analysis (PCA). Positive and negative ion mode PCA score maps **(A,B)** and pathway enrichment analyses **(C,D)** of larva, pupa, and adult groups are shown. Different colors in the **(A,B)** represented different groups and each point represented a sample.

To obtain comprehensive information on the metabolome, small molecule metabolites in the larval, pupal, and adult samples were analyzed by UPLC-Q-TOF-MS. A preliminary analysis of all samples was performed by PCA. The larva and adult groups were biased compared with the pupa collection in both positive and negative ion maps, whereas the larva and adult groups were overlapped (Figures 2A,B).

By OPLS-DA model, 43 potential aromatic biomarkers were screened in positive ion mode. Out of them, a subset of 39 was greater in the pupal specimen compared with those in the larval sample, while another subset of 40 was more in the pupal specimen than those in the adult group (Table 1). A total of 11 potential aromatic biomarkers were obtained in negative ion mode. Among them, subsets of 9 and 8 were greater in the pupal sample than those in the larva and adult groups, respectively (Table 2).

**Table 1:** Differential xenobiotics with higher levels in pupae v.s. larvae/adults in positive ion mode.

**KEGG CID**	**Metabolites**	**P/L**	**P/A**	**VIP(P/L)**	**P(P/L)**	**VIP(P/A)**	**P(P/A)**
C00755	Vanillin	↑	↑	2.2	<0.01	2.1	<0.01
C02519	4-Methoxybenzoate	↑	↑	2.2	<0.01	2.1	<0.01
C16472	3-Fluorocatechol	↑	↑	4.4	<0.01	5.2	<0.01
C16473	4-Fluorocatechol	↑	↑	4.4	<0.01	5.2	<0.01
C14110	4-Hydroxymethylcatechol	↑	↑	2.2	<0.01	2.1	<0.01
C02909	(2-Naphthyl)methanol	↑	↑	4.7	<0.01	4.8	<0.01
NA	2-Methoxynaphthalene	↑	↑	4.7	<0.01	4.8	<0.01
C14790	1-Naphthylamine		↑			3.3	<0.01
C00601	Phenylacetaldehyde	↑	↑	2.4	<0.01	2.5	<0.01
C04043	3,4-Dihydroxyphenylacetaldehyde	↑	↑	2.2	<0.01	2.1	<0.01
C02505	2-Phenylacetamide		↑			2.0	<0.01
C02083	Styrene oxide	↑	↑	2.4	<0.01	1.5	<0.01
C05627	4-Hydroxystyrene	↑	↑	2.4	<0.01	1.5	<0.01
C06758	4-Methylbenzaldehyde	↑	↑	2.4	<0.01	1.5	<0.01
C07209	3-Methylbenzaldehyde	↑	↑	2.4	<0.01	1.5	<0.01
C07214	2-Methylbenzaldehyde	↑	↑	2.4	<0.01	1.5	<0.01
C02519	p-Anisic acid	↑	↑	2.2	<0.01	2.1	<0.01
C07113	Acetophenone	↑	↑	2.4	<0.01	1.5	<0.01
C03663	2′,4′-Dihydroxyacetophenone	↑	↑	2.2	<0.01	2.1	<0.01
C10675	3′,4′-Dihydroxyacetophenone	↑	↑	2.2	<0.01	2.1	<0.01
C13635	2,4′-Dihydroxyacetophenone	↑	↑	2.2	<0.01	2.1	<0.01
C15513	Benzyl acetate		↑			2.0	<0.01
NA	4-Hydroxy-3-methylbenzoic acid	↑	↑	2.2	<0.01	2.1	<0.01
C10804	2-(Hydroxymethyl)benzoic acid	↑	↑	2.2	<0.01	2.1	<0.01
C02181	Phenoxyacetic acid	↑	↑	2.2	<0.01	2.1	<0.01
NA	3-Cresotinic acid	↑	↑	2.2	<0.01	2.1	<0.01
C14103	4-Methylsalicylate	↑	↑	2.2	<0.01	2.1	<0.01
D07721	Chlophedianol	↑		1.2	<0.01		
NA	Hericenone A	↑	↑	2.5	<0.01	2.4	<0.01
C07311	Stanozolol	↑	↑	3.4	<0.01	3.6	<0.01
C10794	Ginkgoic acid	↑	↑	3.2	<0.01	4.8	<0.01
NA	Methyl-[10]-shogaol	↑	↑	3.2	<0.01	4.8	<0.01
C07420	Pentamidine	↑	↑	1.6	<0.01	1.5	<0.01
C08157	Testosterone enanthate	↑	↑	1.2	<0.01	1.2	<0.01
NA	Pregnanetriol	↑	↑	3.7	<0.01	3.9	<0.01
C14606	11-Hydroxyandrosterone	↑		1.1	0.0145		
NA	Saponin H	↑	↑	1.5	<0.01	1.4	<0.01
NA	Assamsaponin D	↑	↑	1.5	<0.01	1.4	<0.01
NA	2,3-Dihydrobenzofuran	↑	↑	2.4	<0.01	2.5	<0.01
C11168	Tetrabenazine	↑	↑	1.6	<0.01	1.5	<0.01
C12508	Nateglinide	↑	↑	1.6	<0.01	1.5	<0.01
NA	Eremopetasinorol	↑		1.7	<0.01		
NA	Chloropanaxydiol	↑		1.0	<0.01		

*P/L, pupal group vs. larval group; P/A, pupal group v.s. adult group; ↑, upregulation*.

**Table 2:** Differential xenobiotics with higher levels in pupae v.s. larvae/adults in negative ion mode.

**KEGG CID**	**Metabolites**	**P/L**	**P/A**	**VIP(P/L)**	**P(P/L)**	**VIP(P/A)**	**P(P/A)**
NA	4-Hydroxybenzylamine		↑			1.2	<0.01
C10770	5-(8-Pentadecenyl)-1,3-benzenediol	↑	↑	1.3	<0.01	1.9	0.016
C03719	Phenylacetothiohydroximate	↑	↑	1.8	<0.01	1.7	<0.01
C08158	Testosterone propionate	↑	↑	5.9	<0.01	5.3	<0.01
C14643	Medrysone	↑	↑	5.9	<0.01	5.3	<0.01
C08185	Triamcinolone hexacetonide	↑	↑	1.7	0.02	1.6	0.02
C07119	Medroxyprogesterone		↑			5.3	<0.01
C01780	Aldosterone	↑		1.0	<0.01		
C18039	Aldosterone hemiacetal	↑		1.0	<0.01		
C19873	26-Hydroxycastasterone		↑			1.7	<0.01
C19326	p-Anisidine	↑	↑	1.5	0.01	1.2	<0.01
C07369	Prednisolone	↑		1.0	0.01		

*P/L, pupal group vs. larval group; P/A, pupal group v.s. adult group; ↑, upregulation*.

### Active Pathway in Pupae

Biological pathway analysis revealed that five active pathways were involved in the metabolism of aromatics in pupae compared with those in the larva/adult groups. These ways contained the catabolism of styrene, naphthalene, fluorobenzoate, aminobenzoate, and benzoate, respectively (Figures 2C,D).

### Enrichment of Xenobiotics in Aposymbiontic Pupa

**Figure 3:**
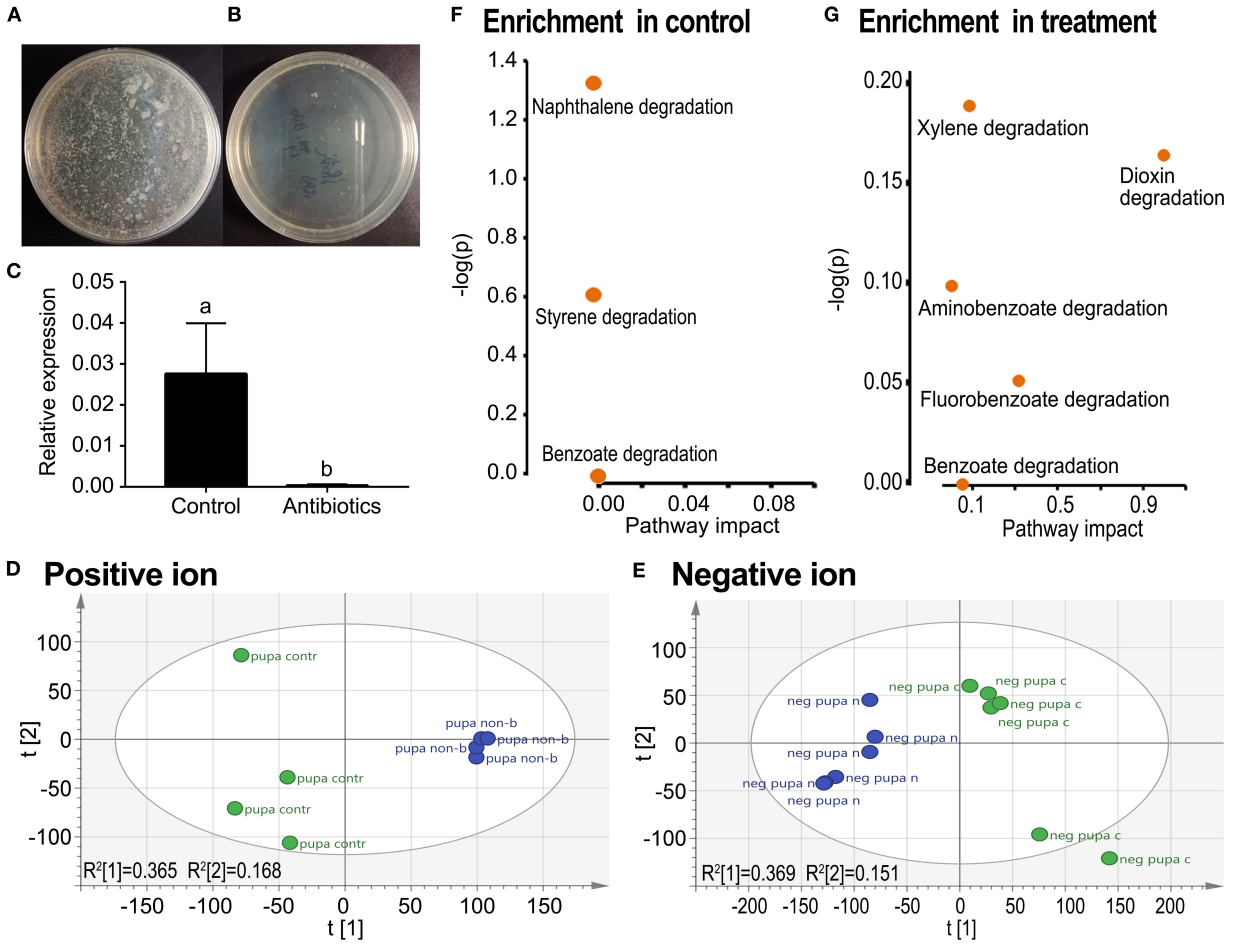
Grouping comparison of metabolomics in control and antibiotic-fed pupae in *L. decemlineata*. The removal of bacteria was examined by plate culture **(A,B)** and qRT-PCR using a pair of universal primers of 16S rDNA from the Domain Bacteria **(C)**. Relative expression level of 16S rDNA was calculated by the 2^−ΔΔCT^ method. Different letters indicate significant difference at *p*-value < 0.05 using analysis of variance with the Tukey–Kramer test. The UPLC-Q-TOF-MS resultant data were analyzed by PCA. Positive and negative ion mode PCA score maps **(D,E)** and pathway enrichment analyses **(F,G)** in control vs. antibiotic-fed pupal samples are shown. Green and blue colors in the **(D,E)** represented control and aposymbiontic pupal groups and each point represented a sample.

Feeding a mixture of three antibiotics by the fourth-instar larvae almost completely removed culturable aerobic bacteria (Figure 3B vs. Figure 3A), and all other bacteria (Figure 3C) in the resultant pupae. In the PCA graphs, the aposymbiontic groups were biased compared with the control groups in both positive and negative ion maps (Figures 3D,E).

In both positive and negative ion modes, the differences in xenobiotic biomarkers between control and aposymbiontic pupal samples included a great number of aromatic compounds (Tables 3, 4).

**Table 3:** Differential xenobiotic and other secondary metabolite biomarkers with higher levels in controls than antibiotic-fed pupae in positive ion mode.

**KEGG CID**	**Metabolites**	**C/T**	**VIP**	***P***
C06758	4-Methylbenzaldehyde	↑	3.0	<0.01
C07209	3-Methylbenzaldehyde	↑	3.0	<0.01
C07214	2-Methylbenzaldehyde	↑	3.0	<0.01
C05775	N1-(alpha-D-ribosyl)-5,6-dimethylbenzimidazole	↑	1.5	<0.01
C06846	Benztropine	↑	1.4	<0.01
NA	2,3-Dimethylbenzofuran	↓	1.3	<0.01
NA	4-(1-Methylethenyl)benzaldehyde	↓	1.3	<0.01
C06578	4-Isopropylbenzoic acid	↓	1.5	<0.01
C00601	Phenylacetaldehyde	↑	3.0	<0.01
C05332	Phenylethylamine	↑	1.1	0.02
C02455	1-Phenylethylamine	↑	1.1	0.02
C12288	alpha-Amylcinnamaldehyde	↑	3.0	<0.01
C02083	Styrene oxide	↑	3.0	<0.01
C07083	Styrene	↓	1.4	0.03
C05627	4-Hydroxystyrene	↑	3.0	<0.01
C01455	Toluene	↓	1.5	0.01
C07113	Acetophenone	↑	3.0	<0.01
C02909	(2-Naphthyl)methanol	↑	2.0	<0.01
NA	2-(2-Methylpropoxy)naphthalene	↑	2.4	0.012
C12303	Phenethyl acetate	↓	1.5	<0.01
NA	2-Phenyl-2-butenal	↓	1.3	<0.01
NA	4-Phenyl-2-butenal	↓	1.3	<0.01
NA	4-(1-Methylethenyl)benzaldehyde	↓	1.3	<0.01
NA	2-Methyl-3-phenyl-2-propenal	↓	1.3	<0.01
NA	3-(4-Methylphenyl)-2-propenal	↓	1.3	<0.01
C08155	Nandrolone phenpropionate	↓	3.0	0.011
NA	2,3-Dimethylbenzofuran	↓	1.3	<0.01
C10453	Eugenol	↓	1.5	<0.01
C10469	Isoeugenol	↓	1.5	<0.01
C10469	trans-Isoeugenol	↓	1.5	<0.01
C15520	7α,25-Dihydroxycholesterol	↓	2.0	<0.01
C06341	7α,27-Dihydroxycholesterol	↓	2.0	<0.01
C15518	7α,24S-Dihydroxycholesterol	↓	2.0	<0.01
C05453	7α,12α-Dihydroxy-5β-cholestan-3-one	↓	2.0	<0.01
C05458	7α,12α-Dihydroxy-5α-cholestan-3-one	↓	2.0	<0.01
NA	(3α,17α,23S)-17,23-Epoxy-3,29-dihydroxy-27-norlanost-8-en-24-one	↓	2.7	<0.01
NA	γ-Chaconine	↑	1.2	<0.01

*C/T, pupal control group v.s. pupae treated with antibiotics group; ↑, upregulation; ↓, downregulation*.

**Table 4:** Differential xenobiotics with higher levels in controls than antibiotic-fed pupae in negative ion mode.

**KEGG CID**	**Metabolites**	**P/L**	**VIP**	***P***
C01408	Benzoin	↑	1.4	<0.01
NA	Benzosimuline	↑	1.9	<0.01
NA	Phenylmethyl benzeneacetate	↑	1.1	<0.01
NA	2-Phenylethyl benzoate	↑	1.1	<0.01
C13632	4,4′-Dihydroxy-α-methylstilbene	↑	1.1	<0.01
C09814	Benzonitrile	↓	1.4	<0.01
C07178	Trimethobenzamide	↓	2.8	<0.01
C02351	1,2-Benzoquinone	↓	1.7	<0.01
NA	N-[2-(4-Hydroxyphenyl)ethyl]benzamide	↓	1.1	<0.01
C07527	Benzocaine	↓	1.1	<0.01
C12537	Benzyl benzoate	↑	1.4	<0.01
C15513	Benzyl acetate	↓	1.0	<0.01
C02351	1,2-Benzoquinone	↓	1.7	<0.01
C00230	3,4-Dihydroxybenzoic acid	↓	1.7	<0.01
C00196	2,3-Dihydroxybenzoic acid	↓	1.7	<0.01
NA	2,6-Dihydroxybenzoic acid	↓	1.7	<0.01
NA	3,5-Dihydroxybenzoic acid	↓	1.7	<0.01
NA	2-Dodecylbenzenesulfonic acid	↓	9.6	<0.01
C06433	5′-Benzoylphosphoadenosine	↑	1.7	0.014
C04221	trans-1,2-Dihydrobenzene-1,2-diol	↓	2.4	<0.01
C10812	3,4-Methylenedioxybenzaldehyde	↓	1.1	<0.01
C03574	2-Formylaminobenzaldehyde	↓	1.4	<0.01
NA	N-Undecylbenzenesulfonic acid	↓	10.2	<0.01
NA	2-Dodecylbenzenesulfonic acid	↓	9.6	<0.01
NA	N1-(2,4-Dimethoxybenzyl)-n2-(2-(pyridin-2-yl) ethyl)oxalamide	↑	1.4	<0.01
NA	N1-(2-Methoxy-4-methylbenzyl)-n2-(2-(pyridin-2-yl) ethyl)oxalamide	↓	1.2	<0.01
NA	N1-(2-Methoxy-4-methylbenzyl)-n2-(2-(5-methylpyridin-2-yl)ethyl)oxalamide	↑	1.2	<0.01
NA	α-(Methoxyimino)-N-methyl-2-[[[1-[3-(trifluoromethyl) phenyl]ethoxy]imino]methyl]benzeneacetamide	↓	1.1	<0.01
C05775	N1-(α-D-ribosyl)-5,6-dimethylbenzimidazole	↑	1.1	<0.01
C06433	5′-Benzoylphosphoadenosine	↑	1.7	0.014
C02372	4-Aminophenol	↑	1.1	<0.01
C01987	2-Aminophenol	↑	1.1	<0.01
C08061	2′-Aminobiphenyl-2,3-diol	↓	2.6	<0.01
NA	3-Pentadecylphenol	↓	1.1	<0.01
NA	Ethyl 4-phenylbutanoate	↓	2.0	<0.01
C02137	Phenylglyoxylic acid	↓	1.1	<0.01
NA	4-Phenyl-2-butyl acetate	↓	2.0	<0.01
NA	2-Methyl-1-phenyl-2-propanyl acetate	↓	2.0	<0.01
NA	(Z)-3-Phenyl-2-propenal	↑	1.1	<0.01
C06746	N-(2-Phenylethyl)-acetamide	↓	1.5	<0.01
C07734	2-Hydroxy-6-oxo-6-(2-hydroxyphenoxy)-hexa-2,4-dienoate	↓	1.1	<0.01
D05095	Mycophenolate sodium	↑	1.7	<0.01
NA	Phenethylamine glucuronide	↑	1.3	<0.01
C07437	Phensuximide	↑	1.8	<0.01
C04468	(+)-cis-3,4-Dihydrophenanthrene-3,4-diol	↑	1.4	<0.01
NA	2-Phenylethyl β-D-glucopyranoside	↑	2.3	<0.01
C07440	Phenylbutazone	↑	1.9	<0.01
NA	7-(4-Hydroxyphenyl)-1-phenyl-4-hepten-3-one	↑	1.2	<0.01
C07911	Phenylpropanolamine	↑	1.1	<0.01
NA	(Z)-3-Phenyl-2-propenal	↑	1.1	<0.01
NA	Phenylmethyl benzeneacetate	↑	1.1	<0.01
C03719	Phenylacetothiohydroximate	↑	5.3	<0.01
NA	2-Phenylethyl benzoate	↑	1.1	<0.01
NA	1-(5-Acetyl-2-hydroxyphenyl)-3-methyl-1-butanone	↑	1.4	<0.01
C18043	Cholesterol sulfate	↑	6.2	<0.01
C00477	Ecdysone	↑	1.4	<0.01
C02633	20-Hydroxyecdysone	↑	1.4	<0.01
C02513	3-Dehydroecdysone	↑	1.1	<0.01
NA	16-Oxoestrone	↑	2.1	<0.01
C00468	Estrone	↑	1.9	<0.01
C02537	17α-Estradiol	↑	4.4	<0.01
C05302	2-Methoxyestradiol	↑	4.4	<0.01
C05301	2-Hydroxyestradiol	↑	1.5	<0.01
C14209	4-Hydroxyestradiol	↑	1.5	<0.01
C05295	19-Oxotestosterone	↑	4.4	<0.01
NA	Zapotin	↑	1.7	<0.01
C05485	21-Hydroxypregnenolone	↓	3.8	<0.01
C18038	7α-Hydroxypregnenolone	↓	3.8	<0.01
C06390	16α-Hydroxypregnenolone	↓	3.8	<0.01
C05138	17α-Hydroxypregnenolone	↓	3.8	<0.01
C04518	(20S)-17,20-Dihydroxypregn-4-en-3-one	↓	3.8	<0.01
C18040	5α-Dihydrodeoxycorticosterone	↓	3.8	<0.01

*C/T, pupal control group v.s. pupae treated with antibiotics group; ↑, upregulation; ↓, downregulation*.

Metabolic pathway analysis revealed that a total of seven active pathways were enriched in either control or aposymbiontic pupal samples (Figures 3F,G). These ways were involved in the degradation of styrene, naphthalene, benzoate, fluorobenzoate, aminobenzoate, xylene, and dioxin.

### Metabolism Pathways

**Figure 4:**
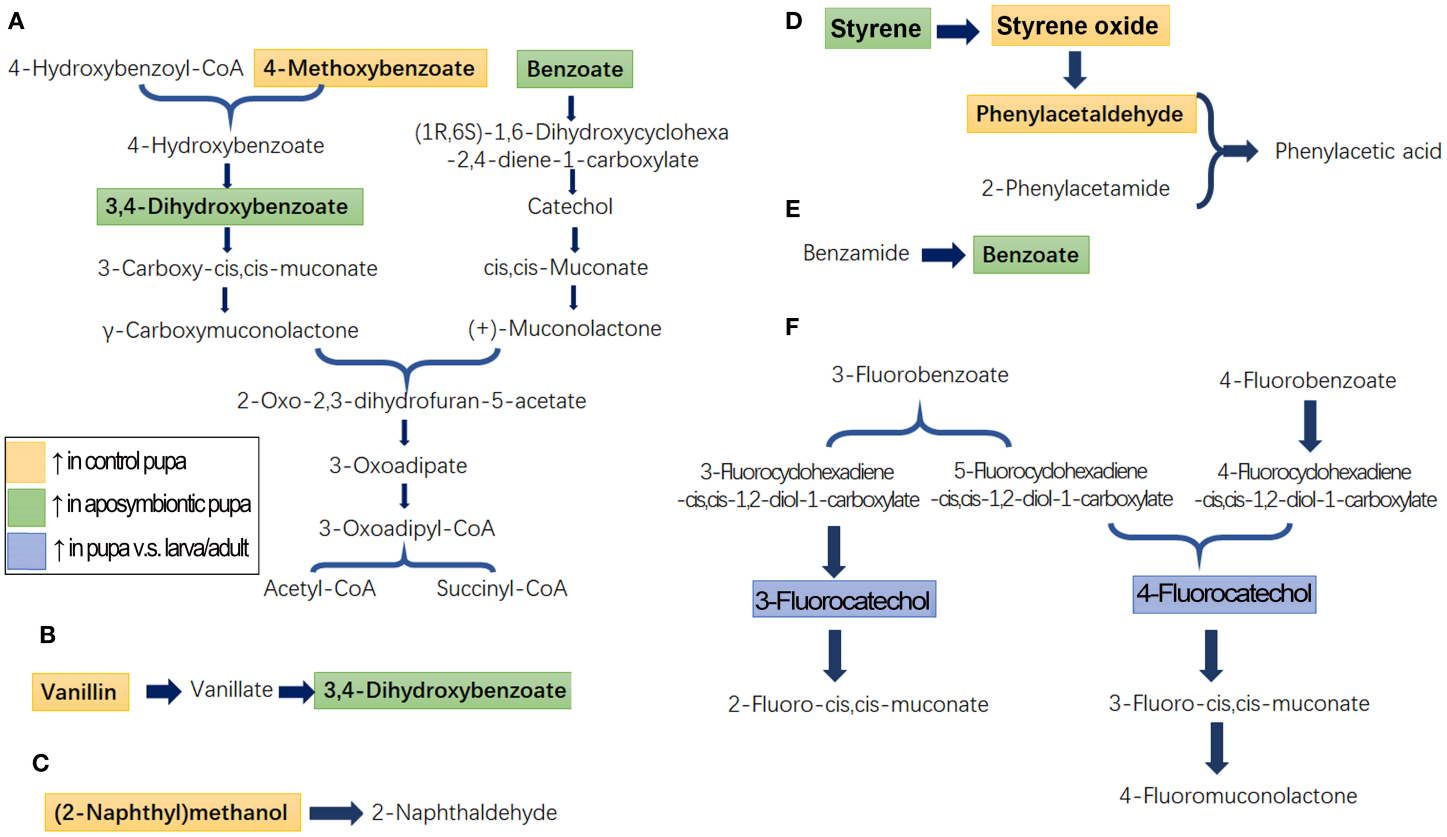
Schematic diagram of the disturbed metabolic pathways in pupae. A total of seven active pathways were enriched in the pupal specimen by KEGG (http://www.kegg.jp) analysis. These ways were associated with the biodegradation of benzoate and 4-methoxybenzoate **(A)**, vanillin **(B)**, naphthalene **(C)**, styrene **(D)**, benzamide **(E)**, and fluorobenzoate **(F)**. The light orange and light green boxes indicate metabolites significantly higher and lower in the control pupal group than in the antibiotic-fed group, respectively. The light blue boxes mark metabolites significantly higher in the pupal group than in the larval and adult samples.

Identified metabolites and biological pathways were imported into the KEGG (http://www.kegg.jp/) to find interactions. Among networks for catabolism of aromatics, benzoates, 4-methoxybenzoate, vanillin, and benzamide were finally transferred into succinyl-CoA and acetyl-CoA, which were completely degraded to CO_2_ through tricarboxylic acid cycle (Figures 4A,B,E). Fluorobenzoates, for instance, 3-fluorobenzoate and 4-fluorobenzoate, were finally converted to 2-fluoro-cis, cis-muconate, 3-fluoro-cis, cis-muconate, and 4-fluoromuconolactone (Figure 4F). Styrene was oxidized to phenylacetic acid (Figure 4D). In contrast, the degradation of (2-naphthyl)methanol (naphthalene) was only partially annotated by KEGG (Figure 4C).

Comparison of metabolite enrichment between control and aposymbiontic pupal samples revealed that 4-methoxybenzoate (Figure 4A), vanillin (Figure 4B), (2-naphthyl)methanol (Figure 4C), styrene oxide, and phenylacetaldehyde (Figure 4D) were higher in the control group. These findings indicated that the soil aromatic precursors could be actively converted into these compounds by pupa bacteria. Conversely, benzoate, styrene, and 3,4-dihydroxybenzoate were accumulated in the aposymbiontic pupal group (Figures 4A,B,E). These results suggested that pupa bacteria were associated with the catabolism of the three chemicals. In contrast, the contents of 3-fluorocatechol and 4-fluorocatechol in control group were similar to those in the aposymbiontic pupal group (Figure 4F). These data implied that pupae may metabolize fluorobenzoates.

## Discussion

To the best of our knowledge, the stage-dependent alterations in the biodegradation of aromatics by the stage-specific bacteria flora in insects have not been well-explored. In the present study, PCA analysis of UPLC-Q-TOF-MS data revealed that the larva and adult groups were biased compared with the pupa collection in both positive and negative ion maps (Figure 2). The shift indicates that the metabolites in the pupa group differ from those in the larva and adult groups. In accordance with the indication, the habitat in which the larvae and adults live is different from that of pupae, with the larva and adult settling on potato plants and the pupa in soil (Meng et al., 2019).

### The Pupae Mainly Rely on Bacteria to Biodegrade Aromatics

Incorporation of plant residues and organic fertilizers into soil brings about some secondary chemicals, e.g., alkaloids, terpenoids, cardenolides, glucosinolates, and oxalates (Zhang et al., 2020), and their metabolites such as monoaromatics (e.g., BTEX and phenol) and polycyclic aromatics (e.g., naphthalene, dioxin, and steroids) (Chen et al., 2017; Steinmetz et al., 2019). These compounds often exert deleterious effects to soil-dwelling pupae when accumulated to high concentrations within the bodies. Insects are hypothesized to specify their microbial community compositions to degrade these substances to avoid intoxication, if suitable ecological conditions are satisfied (Zhang et al., 2020). However, the experimental evidence to support the hypothesis is very limited.

In the present study, we analyzed the bacterial OTU data (Kang et al., 2021) by KEGG and found that a subset of bacterial genes was abundantly expressed at the pupal stage. These genes were associated with the catabolism of aromatics (Figure 1). Consistently, metabolomic analysis displayed that dozens of monoaromatics, polycyclic aromatics, and steroids were richer in the pupal sample than those in the larval and adult specimens (Tables 1, 2).

According to the identified metabolites and biological pathways, a total of seven active ways were enriched. These active pathways are mainly involved in the degradation of various benzoates and their precursors, which include benzoate, 4-methoxybenzoate, 3-fluorobenzoate, 4-fluorobenzoate, vanillin (aminobenzoate), and benzamide (Figure 4). Most of these benzoates were finally transferred into succinyl-CoA and acetyl-CoA, which were completely degraded to CO_2_ through tricarboxylic acid cycle. Similarly, *Dechloromonas* sp. strains RCB and JJ can completely break down aromatic compounds into CO_2_, coupled with the reduction of nitrate (Coates et al., 2001). *Dechloromonas* belongs to proteobacteria. Among the 18 pupa-specific genera identified (Kang et al., 2021), *Escherichia_Shigella, Acinetobacter, Pseudomonas, Lysobacter*, and *Stenotrophomonas* are also proteobacteria. One or several genera listed above may be responsible for the catabolism of the benzoates in *L. decemlineata* pupae.

Specifically, we uncovered that pupa bacteria were involved in the aerobic degradation of styrene to phenylacetic acid in *L. decemlineata* (Figure 4). In agreement with our result, the way in other documented results involves epoxidation of the vinyl side chain, followed by isomerization of the epoxy styrene to form phenylacetaldehyde. This compound is subsequently oxidized to phenylacetic acid through the action of either an NAD^+^- or phenazine methosulfate-dependent dehydrogenase (O'Connor and Dobson, 1996; O'Leary et al., 2002). Conversion of styrene to phenylacetic acid has been documented in various bacterial strains; examples include *Pseudomonas putida* CA-3 (O'Connor et al., 1995), *P. fuorescens* ST (Marconi et al., 1996), *Pseudomonas* sp. VLB120 (Panke et al., 1999), *Pseudomonas* sp. Y2 (Utkin et al., 1991; Velasco et al., 1998), *Xanthobacter* sp. 124X (Hartmans et al., 1989), and *Xanthobacter* sp. S5 (Hartmans et al., 1990). *Pseudomonas* is among the 18 pupa-specific genera identified recently (Kang et al., 2021), and the genus may be responsible for the catabolism of styrene in *L. decemlineata* pupae.

At present, polycyclic aromatic hydrocarbons produced by all vertebrates as well as some invertebrates have been considered one of the most important environmental problems (Xiong et al., 2019; Chiang et al., 2020). In this study, we discovered that the polycyclic aromatic hydrocarbon (2-naphthyl)methanol (naphthalene) can be biodegraded by pupa-specific bacteria in *L. decemlineata* (Figure 4). It has been reported that naphthalene can be catabolized by *P. fluorescens* AH-40 (Mawad et al., 2020), *P. putida* BS3701 (Pozdnyakova-Filatova et al., 2020), and *Stenotrophomonas* sp. S1VKR-26 (Patel and Patel, 2020). Naphthalene is converted *via* salicylate and catechol to the intermediates of tricarboxylic acid cycle in *P. putida* PpG1 (Yen and Gunsalus, 1982), catalyzed by four key enzymes, namely, naphthalene 1,2-dioxygenase, salicylate hydroxylase, catechol 2,3-dioxygenase, and catechol 1,2-dioxygenase (Izmalkova et al., 2006). In this survey, we determined that 3- and 4-fluorocatechol, 4-hydroxymethylcatechol, and 4-methylsalicylate were higher in the pupae than those in the larvae and adults. It appears that the same naphthalene transformation way is present in the *L. decemlineata* pupae. Consistent with the metabolomic result, our microbiome analysis revealed that both *Pseudomonas* and *Stenotrophomonas* are among the 18 pupa-specific genera identified recently (Kang et al., 2021).

Both biogenic (natural) and anthropogenic steroids are frequently detected in soils and aquatic environments in China (Chiang et al., 2020). For example, oestrogens, androgens, progestogens, glucocorticoids, and mineralocorticoids are detected in the surface water of urban rivers in Beijing (Chang et al., 2009). Bacteria are responsible for mineralizing polycyclic aromatic hydrocarbons from the biosphere (Chiang et al., 2020). Among 18 pupa-specific bacterial genera (Kang et al., 2021), *Nocardia* (Coombe et al., 1966), *Rhodococcus* (Fernandez de las Heras et al., 2009; Li et al., 2018), and *Stenotrophomonas* (Juhasz et al., 2000; Tachibana et al., 2003; Guan et al., 2019; Xiong et al., 2019) have been documented to degrade steroids from other environments. Whether the three bacterial genera in *L. decemlineata* can break down steroids deserves further research.

Saponins are a class of secondary plant metabolites which includes triterpenoids, steroids, and steroidal alkaloids glycosylated with one or more sugar chains. They are produced by many plant species (Zhang et al., 2020). Saponins provoke molting defects in, and exert deleterious effects on insects (De Geyter et al., 2007; Podolak et al., 2010; Cai et al., 2016; Dolma et al., 2017). Therefore, tea saponin has been widely used as an insecticide in China (Cai et al., 2016). We herein demonstrated that the contents of saponin H and assamsaponin D were higher in the pupae compared with those in the larvae and adults in *L. decemlineata* (Figure 4).

Although a considerable amount of saponins is indicated in the soil, *L. decemlineata* pupae could still develop into adults, indicating that other factors may help *L. decemlineata* resist saponins. Some bacteria, for instance, *Acinetobacter calcoaceticus* and *A. oleivorans*, are known to detoxify saponins (Zhang et al., 2020). Moreover, the mixed cultures of *Methanobrevibacter* spp. and *Methanosphaera stadtmanae* in the crop of the avian foregut fermenter in *Opisthocomus hoazin* are able to reduce the hemolytic activity of Quillaja saponins by 80% within a few hours (García-Amado et al., 2007). Consistent with these results, *Acinetobacter* is a pupa-specific genus (Kang et al., 2021), and it may be responsible for the metabolism of saponins in the *L. decemlineata* pupae.

### Elimination of Aromatics in the Larvae and Adults

Potato plants contain many aromatics, where some exert noxious effects when accumulated to high concentrations within insect bodies (Gandia-Herrero and Garcia-Carmona, 2013; Kostyn et al., 2020). In this survey, we discovered that only 6 and 7 aromatics were accumulated in the larvae and adults, respectively, in contrast to 42 cumulated aromatics in the pupae (Tables 1, 2). The less accumulation implies the more active elimination of aromatics in the *L. decemlineata* larvae and adults.

Consistently, the microbiota are widely distributed in the larval or/and adult guts (Jing et al., 2020). These gut microbiota are involved in the breakdown of noxious compounds in numerous insect species in Coleoptera (Ceja-Navarro et al., 2015; Berasategui et al., 2017; Zhang et al., 2020), Lepidoptera (Vilanova et al., 2016; Zeng et al., 2020), Diptera (Griffin and Reed, 2020), Hymenoptera (Wu et al., 2020), and Isoptera (Van Dexter and Boopathy, 2019).

In this survey, the bacterial OTU data (Kang et al., 2021) revealed that the catabolism of aromatics was less active in the larvae and adults compared with that in *L. decemlineata* pupae (Figure 1). Therefore, the bacterial biodegradation of aromatics only partially contributes to the removal of excessive aromatics. Intestinal excretion should be another route to eliminate superfluous aromatics (Rozman, 1985). In fact, insecticides can be excreted by insects, directly or indirectly (modified forms) (Quesada et al., 2020). In *L. decemlineata* larvae and adults, superfluous aromatics in food may be excreted through the guts directly, or transferred to more hydrophilic forms. Only those potato aromatics absorbed by *L. decemlineata* larvae and adults need to be biodegraded by bacteria. Conversely, the alimentary canal is not well-developed in *L. decemlineata* pupae and cannot actively remove excessive aromatics. The pupae mainly depend on bacteria to catabolize the noxious substances.

In summary, we uncovered the stage-dependent alterations in bacterial degradation of aromatics in *L. decemlineata*. The candidate bacterial genera contributing to aromatic catabolism were *Nocardia, Rhodococcus, Enterococcus, Acinetobacter, Pseudomonas*, and *Stenotrophomonas*, among others. This study provides new insights into the adaptation of *L. decemlineata* to different environmental niches and offers a better understanding of the relationship between ONS and a shift of bacterial flora. Moreover, since removal of the symbiotic bacteria inhibited the breakdown of superfluous aromatics (this study) and results in a decrease in the emergence rate and adult weight (Kang et al., 2021), disruption of bacterial communities may be a potential strategy to control *L. decemlineata*.

## Data Availability Statement

The datasets presented in this study can be found in online repositories. The names of the repository/repositories and accession number(s) can be found in the article/supplementary material.

## Author Contributions

W-NK, LJ, H-YM, and G-QL conceived the study, and participated in the design of the experiments and the interpretation of the results. W-NK, LJ, and H-YM performed the experiments. W-NK, LJ, and G-QL wrote the first draft of the manuscript. All authors contributed to the article and approved the submitted version.

## Funding

This research was supported by the National Natural Science Foundation of China (32072416), and the China Agriculture Research System of MOF and MARA (CARS-09-P22).

## Conflict of Interest

The authors declare that the research was conducted in the absence of any commercial or financial relationships that could be construed as a potential conflict of interest.

## Publisher's Note

All claims expressed in this article are solely those of the authors and do not necessarily represent those of their affiliated organizations, or those of the publisher, the editors and the reviewers. Any product that may be evaluated in this article, or claim that may be made by its manufacturer, is not guaranteed or endorsed by the publisher.
